# Hand-book of the Diseases of the Nervous System

**Published:** 1887-04

**Authors:** 


Handbook of the Diseases of the Nervous System. *By* Joseph P. Ross, M.D., LL.D. *Large octavo, pp. xx, 723. Philadelphia :* Lea Brothers & Company. *Chicago :* A. C. McClurg & Company. 1885.

Students, as well as practitioners, will find in this book an excellent compendium of the diseases of the nervous system.

The work is divided into two parts. The first seven chapters, comprising 234 pages, are devoted to the consideration of the anatomy and physiology of the nervous system, together with a short chapter on general treatment. The remaining part is devoted to the special diseases of the nervous system.

Dr. Ross, in his larger work, "Treatise on Diseases of the Nervous System," has shown himself to be a thorough master and teacher of neurology. The present work fully bears out the high estimate one is inclined to place on any work bearing his name. The attempt to formulate the chief facts in neurology, from the simplest expression of nervous function to the highest psychical manifestations of which the human brain is capable, and to do this within the compass of an ordinary handbook, is a task worthy of the keenest intellect. The work of Dr. Ross, as a whole, marks a distinct step forward in neurological literature. He has to a large extent abandoned some of the older theories, and he approaches the subject purely from the standpoint of the evolutionist. This is clearly outlined in his classification, and the treatment of the different subjects.

The work is concisely and clearly written. Special mention is due the publishers for the handsome manner in which the work is issued. H. N. M.

